# MSL: Facilitating automatic and physical analysis of published scientific literature in PDF format

**DOI:** 10.12688/f1000research.7329.3

**Published:** 2018-04-04

**Authors:** Zeeshan Ahmed, Thomas Dandekar

**Affiliations:** 1 Genetics and Genome Sciences, School of Medicine, University of Connecticut Health Center, Farmington, CT, 06032, USA; 2 Institute for Systems Genomics, University of Connecticut Health Center, Farmington, CT, 06032, USA; 3 Department of Bioinformatics, Biocenter, University of Wuerzburg, Wuerzburg, 97074, Germany

**Keywords:** Bioinformatics, Data mining, Images, Scientific literature, Text, OCR, PDF, Biomedical

## Abstract

Published scientific literature contains millions of figures, including information about the results obtained from different scientific experiments e.g. PCR-ELISA data, microarray analysis, gel electrophoresis, mass spectrometry data, DNA/RNA sequencing, diagnostic imaging (CT/MRI and ultrasound scans), and medicinal imaging like electroencephalography (EEG), magnetoencephalography (MEG), echocardiography  (ECG), positron-emission tomography (PET) images. The importance of biomedical figures has been widely recognized in scientific and medicine communities, as they play a vital role in providing major original data, experimental and computational results in concise form. One major challenge for implementing a system for scientific literature analysis is extracting and analyzing text and figures from published PDF files by physical and logical document analysis. Here we present a product line architecture based bioinformatics tool ‘Mining Scientific Literature (MSL)’, which supports the extraction of text and images by interpreting all kinds of published PDF files using advanced data mining and image processing techniques. It provides modules for the marginalization of extracted text based on different coordinates and keywords, visualization of extracted figures and extraction of embedded text from all kinds of biological and biomedical figures using applied Optimal Character Recognition (OCR). Moreover, for further analysis and usage, it generates the system’s output in different formats including text, PDF, XML and images files. Hence, MSL is an easy to install and use analysis tool to interpret published scientific literature in PDF format.

## Introduction

There has been an enormous increase in the amount of the scientific literature in the last decades ^1^. The importance of information retrieval in the scientific community is well known; it plays a vital role in analyzing published data. Most published scientific literature is available in Portable Document Format (PDF), a very common way for exchanging printable documents. This makes it all-important to extract text and figures from the PDF files to implement an efficient Natural Language Processing (NLP) based search application. Unfortunately, PDF is only rich in displaying and printing but requires explicit efforts in the extraction of information, which significantly impacts the search and retrieval capabilities ^2^. Due to this reason several document analysis based tools have been developed for physical and logical document structure analysis of this file type.

PubMed and some other publishing platforms (e.g. DOAJ, Google Scholar) provide search options to locate relevant published manuscripts but do not claims to search over the full-text literature and images. The recently, provided basic information retrieval (IR) system by PubMed is efficient in extracting literature based on published text (titles, authors, abstracts, introduction etc.), with the application of automatic term mapping and Boolean operators ^3^. The normal outcome of a successful NLP and text based query brings a maximum of 20 relevant results per page; however, user can improve the search by customizing the query using the provided advanced options. So far, the current PubMed system, as well many other related system are unable to completely implement an efficient information retrieval system, capable of extracting both text and figures from published PDF files. One of the major and technical challenges is the availability of structured text and figures. To our limited knowledge, there still is no single tool available which can efficiently perform both physical and logical structure analysis of all kinds of PDF files and can extract and classify all kinds of information (embedded text from all kinds of biological and scientific published figures). Different commercial and free downloadable software applications provide support in extracting the text and images from PDF files:

A-PDF ( http://www.a-pdf.com/image-extractor/),

PDF Merge Split Extract ( http://www.pdf-technologies.com/pdf-library-merge-split.aspx),

BePDF ( http://haikuarchives.github.io/BePDF/), KPDF ( https://kpdf.kde.org),

MuPDF ( http://mupdf.com), Xpdf tool ( http://www.foolabs.com/xpdf/),

Power PDF ( http://www.nuance.com/for-business/imaging-solutions/document-conversion/power-pdf-converter/index.htm)

However, these software applications do not provide text and images in a form where they could be considered for further logical analysis e.g. mining text in reading order from double or multiple columns documents (the text of first column followed by the text of second column, and so on), searching marginal text using key-words, removing irrelevant graphics and extracting embedded text inside single and multi-panel complex biological images.

So far, the current PubMed system as well many other related orthodox NLP approaches e.g. 4– 13, are unable to completely implement an efficient information retrieval system, capable of extracting both text and figures from published PDF files.

**Figure 1.**
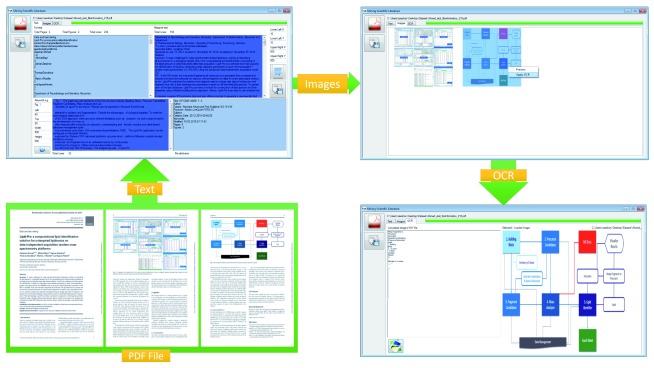
Graphical user interfaces of MSL and modular workflow. This figure shows the graphical user interface and modular workflow of three main components: Text, Image and OCR. A PDF document ^21^ is input and processed by MSL. Text module provides extracted, searched and marginalized text in reading order, and file attributes. Image component provides the preview of extracted images from the document. OCR component provides extracted text from selected and processed image.

**Figure 2.**
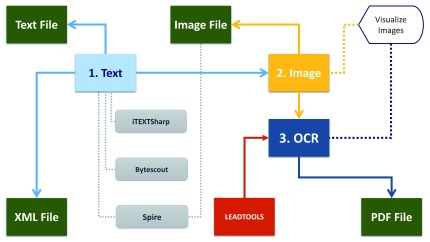
Conceptual architecture of MSL and component’s workflow. This figure shows the conceptual architecture of the MSL application, which consists of three main components: Text, Image and OCR, and nine sub-components: Text File, Image File, Visualize Image, PDF File, LEADTOOLS, XML File, iTEXTSharp, Bytescout, Spire. As figure shows, Text component applies iTEXTSharp, Bytescout, Spire to extract the text from PDF document and write output in XML file. Image components applies Spire to extract images from the PDF document and visualize that using Visualize Image. OCR component applied LEADTOOLS to extract text from images and export that to PDF format.

To meet the technological objectives of this challenge, we took a step forward in the development of a new user friendly, modular and client based system (MSL) for the extraction of full and marginal text from PDF files based on the keywords and coordinates ( Figure 1). Since MSL provides a module for the extraction of figures from PDF files and applies Optical Character Recognizer (OCR) to extract text from all kinds of biomedical and biological Images. MSL comprises three modules working in product-line architecture: Text, Image and OCR ( Figure 2). Each module performs its task independently and its output is used as an input for the next module. It can be configured on Microsoft Windows platforms following a simple six-step installation process.

## Methods

MSL extracts text and figures from the published scientific literature and helps in analyzing embedded text inside figures. The overall methodological implementation and workflow of the MSL is divided into two processes: (I) Text mining and (II) Image analysis. MSL is a desktop application, designed and developed following the scientific software engineering principles of three-layered Butterfly ^14^ software development model.

### Text mining

Physical and logical document analysis is one of the living challenges. To the best of the authors’ knowledge, there is no solution available which can perform efficient physical and logical structural analysis of PDF files, implement completely correct rendering order and classify text in all possible categories e.g. Tile, Abstract, Headings, Figure Captions, Table Captions, Equations, References, Headers, Footers etc.

However, there are some tools available which are helping in this regard e.g. PDF2HTML towards contextual modeling of logical labelling ^15^, PDF-Analyzer for object level document analysis ^16^, XED for hidden structure analysis ^2^, Dolores for the logical structure analysis and recovery ^17^ automatic conversation from PDF to XML ^18^ and PDF to HTML ^19^, Layout-aware ^20^ etc.

We developed MSL’s Text module, which is capable of processing PDF files with single, double or multiple columns. It divides the system’s text based output in four sub-modules: full text, marginal text, keyword based extracted text and file attributes. Full text gives the complete text from PDF file, marginal allows user to give the coordinates (Lower Left X, Lower Left Y, Upper Right X and Upper Right Y) and extract the desired portion of the text from the PDF file. The keyword based text allows user to extract the information from PDF file based on keywords and respective coordinates (Left, Top, Width, Height) e.g. if a user is only interested in getting the figure caption or references, this kind of search will be helpful. The last sub module, File attributes gives the information about input file including title, author, creator, producer, subject, creation date, keywords, modified, number of pages and number of figures.

While implementing Text module, we researched and tried different available commercial and freely downloadable libraries with a focus on full text extraction, marginal text extraction, keyword based text extraction and text extraction from embedded images from PDF files. We tried different implemented systems and libraries ( Table 1) e.g. iTextSharp Bytescout, Spire PDF Sautinsoft PDF Focus Dynamic PDF, PDFBox, iText PDF, QPDF, PoDoFo, Haru PDF Library, JPedal, SVG Imprint, Glance PDF Tool Kit, BCL SharpPDF etc.

**Table 1.** Systems and Libraries tested for MSL. The table gives the list of different systems and libraries, which have been used for the extraction of text from PDF files.

Library Name	Weblink
iTextSharp	( http://sourceforge.net/projects/itextsharp/),
Bytescout	( https://bytescout.com)
Spire PDF	( http://www.e-iceblue.com/Introduce/pdf-for-net- introduce.html)
Sautinsoft PDF Focus	( http://www.sautinsoft.com/products/pdf-focus/)
Dynamic PDF	( https://www.dynamicpdf.com)
PDFBox	( https://pdfbox.apache.org)
iText PDF	( http://itextpdf.com)
QPDF	( http://qpdf.sourceforge.net)
PoDoFo	( http://podofo.sourceforge.net)
Haru PDF Library	( http://libharu.sourceforge.net)
JPedal	( https://www.idrsolutions.com/jpedal/)
SVG Imprint	( http://svgimprint-windows.software.informer.com)
Glance PDF Tool Kit	( http://www.planetpdf.com/forumarchive/53545.asp)
BCL	( http://www.pdfonline.com/corporate/)
SharpPDF	( http://sharppdf.sourceforge.net)

One of the common problems in almost all libraries is merging and mixing of text, using double or multiple columns. Our developed system is the combination of different libraries, useful for different purposes. We have used Spire PDF to remove the Book-marks, iTextSharp for the extraction of full and marginal text, Bytescoute for the keyword based marginalized text search and producing output in the form of XML file ( Figure 2). The generated XML file contains structured (tagged) text along with the information about its coordinates (placement in the file), font (Bold, Italic etc.) and size, which can be used for mapping and pattern recognition tasks.

### Image processing

Image-based analysis is a versatile and inherently multiplexed approach as it can quantitatively measure biological images to detect those features, which are not easily detectable by a human eye. Millions of figures have been published in scientific literature that includes information about results obtained from different biological and medicinal experiments. Several data and image mining solutions have been already implemented, published and are in use in the last 15 years ^22^. Some of the mainstream approaches are towards the analysis of all kinds of images (flow charts, experimental images, models, geometrical shapes, graphs, images of thing or objects, mixed etc.). There are not many approaches proposed for specific kinds of image-analysis e.g. towards the identification and quantification of cell phenotypes ^23^, prediction of subcellular localization of proteins in various organism ^24^, analysis of gel diagrams ^25^, mining and integration of pathway diagrams ^26^.

While implementing a new data-mining tool, one of our goals was to extract images from published scientific literature and try to extract embedded text as well. We analyzed different freely available and commercial OCR systems and libraries including Aspose, PUMA, Microsoft OCR, Tesseract, LEADTOOLS, Nicomsoft OCR, MeOCR OCR, OmniPage, ABBYY, Bytescout claiming to be able to extract embedded text from figures. During our research we found LEADTOOLS ( Figure 2) as one of the best available solutions for this purpose. MSL is capable of automatically extracting images from the PDF files and allowing the user to apply OCR to any extracted image by clicking and enlarging it for a better view (using Windows default image viewer).

## Results and discussion

We tested MSL with similar parameters on randomly selected scientific manuscripts (ten PDF files) from different open access ( *F1000Research, Frontiers, PLOS, Hindawi, PeerJ, BMC*) and restricted access ( *Oxford University Press, Springers, Emerald, Bentham Science, ACM*) publishers, including some of the authors’ published papers, details are given in Table 2. While testing MSL on the selected manuscripts, we observed best overall performance for the manuscripts ^27– 32^, with satisfactory results from almost all publishers (including *Oxford University Press, BMC, Frontiers, PeerJ, Bentham Science, ACM*) in terms of both extracting text in reading order and extracting images. An observed poor performance involved manuscripts from *PLOS* ^33^ *, Hindawi* ^34^, *F1000Research* ^35^ and IEEE ^36^ publishers. Here, in the case of text extraction we observed that the text was in reading order when using manuscripts from *F1000Research* and IEEE but text was without spaces in the manuscript from *PLOS* and with additional lines and extra spaces in the manuscript from *Hindawi*. In the case of figure extraction we observed one common problem among the four manuscripts from these publishers; along with the manuscript images (Figures), embedded journal or publishers’ logos and images were also extracted. Additionally, while analyzing the manuscript from *F1000Research*, we observed that the images were broken into many pieces and it was not possible to find one single complete image. As we did not test all manuscripts from the mentioned publishers, we cannot claim that the results will be the same for all papers from a publisher, as the output may vary in different papers. Our observed results using MSL are given in attached supplementary material ( Supplementary Table S1 and Dataset 1).

Extracted images and text from papers tested using MSL Raw dataset is attached to this manuscript, which categorically provides all images and text in XML format, extracted from manuscripts (from different publishers (included in file names)) using MSL ^37^. Click here for additional data file. Copyright: © 2018 Ahmed Z and Dandekar T 2018 Data associated with the article are available under the terms of the Creative Commons Zero "No rights reserved" data waiver (CC0 1.0 Public domain dedication).

**Table 2.** Papers (PDF files) tested using MSL. The table gives the list of 10 of those manuscripts from different publishers, which have been used for testing and validating the MSL application.

Publishers	DOI	Manuscript Title
*F1000-Research*	10.12688/f1000research.5931.3	Ant-App-DB: a smart solution for monitoring arthropods activities, experimental data management and solar calculations without GPS in behavioral field studies ^35^.
*PLOS*	10.1371/journal.pgen.1006202	The Genomic Aftermath of Hybridization in the Opportunistic Pathogen Candida metapsilosis ^33^.
*Hindawi*	10.1155/2015/723451	Mathematical Properties of the Hyperbolicity of Circulant Networks ^34^.
*IEEE*	10.1109/IC4.2009.4909215	Design implementation of I-SOAS IPM for advanced product data management ^36^.
*BMC*	10.1186/1471-2105-14-218	Software LS-MIDA for efficient mass isotopomer distribution analysis in metabolic modeling ^28^.
*PeerJ*	10.7717/peerj.1319	Anvi’o: an advanced analysis and visualization platform for ‘omics data ^29^.
*Frontiers*	10.3389/fninf.2015.00009	Ontology-based approach for *in vivo* human connectomics: the medial Brodmann area 6 case study ^30^.
*ACM*	10.1145/1838002.1838065	Intelligent semantic oriented agent based search (I-SOAS) ^31^.
*Bentham Science*	10.2174/2213275906666131108211241	DroLIGHT-2: Real Time Embedded and Data Management System for Synchronizing Circadian Clock to the Light-Dark Cycles ^32^.
*Oxford University* *Press*	10.1093/bioinformatics/btu772	Bioimaging-based detection of mislocalized proteins in human cancers by semi-supervised learning ^27^.

**Figure 3.**
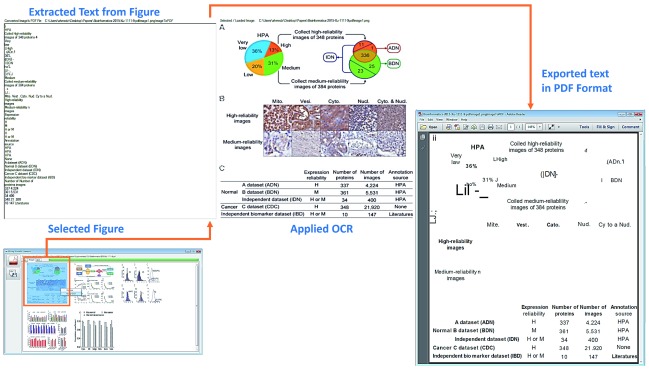
Example: Publication, Figure 1 of (YY *et al.*, 2015). This figure shows document image analysis, text extraction and PDF conversion. A figure (based on three panels; including two charts, one image and a table) is selected from one of the randomly selected papers 2 ^6^. OCR (LEADTOOLS) is applied to extract and report the text from the figure in simple text form (section: Extracted Text from Figure) and in PDF file with similar margins to the original figure (section: Exported text in PDF format).

**Figure 4.**
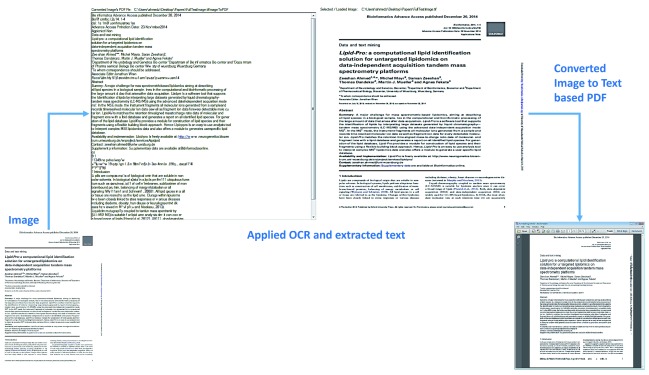
Example: Publication, Page 1 (Ahmed *et al.*, 2015). This figure shows document image analysis, text extraction and PDF conversion. First, scanned image based page of one of the randomly selected papers ^21^ is processed using OCR (LEADTOOLS). Text is extracted from the image and a new PDF is generated, which is based on the text, placed with similar margins to the image file.

To apply MSL, published scientific literature has first to be downloaded in the form of a PDF file, from any published source. The validation process using MSL consists of three major steps: 1) Text mining, 2) Image extraction, and 3) Application of OCR to extract text from selected images as shown in Figure 1, following the implemented workflow as shown in Figure 2. Example results and graphics are shown in Figure 1, Figure 3 and Figure 4. Representation includes the extraction of text and images from one of the randomly selected papers ^21^, and application of OCR to one of the extracted images from another randomly picked publication ^27^.

Figure 1 shows that one randomly selected published article’s PDF file ^21^ is inputted to the MSL’s text, the extracted text is divided into three categories (i) complete text in excellent rendering order (ii) marginalized text and (iii) keyword based searched text. Two figures ( Figure 1 and Figure 2) are extracted and displayed in the image section, and one of those is selected to apply OCR. The applied OCR extracts textual information, which is displayed in and can be exported in a PDF file.

To further validate the application of OCR and discuss different results, Figure 3 show another example of embedded text extraction from a complex figure ^38^, which includes three panels of images (i) colorful pie and circle charts, (ii) biological images and (iii) tabular information. Similar to our prior application of OCR, results are displayed in textual form as well as generated PDF file of extracted text. A noticeable difference between both outputs is that the textual information is presented in line-by-line order whereas in the PDF file the information is displayed in margins with respect to the original image.

The last resultant example is based on the validation of MSL by extracting the textual information from image based PDF files. We produced an image form of one of the randomly selected article ^27^ and then processed one of pages. As Figure 4 shows, the obtained results were comprehensive in both textual as well as the PDF form. This kind of textual extraction can be very helpful, especially when the literature is available in only images e.g. in the case of old published literature in print only format but electronically available in scanned form. MSL produces several files as system output in the parent folder of the files. These files are: XML files (which include structured or tagged information), an Images File (extracted from the PDF file) and PDF files for all analyzed images using OCR. Additionally, extracted text can be split into different IMRAD parts and results can be searched and categorized e.g. based on abstract, introduction, methods, discussion, conclusion and references.

We mentioned earlier that we have tried and implemented different libraries for text and image extraction and analysis. The best text based outcome was observed using iTextSharp, better image extraction was observed using Spire and OCR from LEADTOOLS was the most promising. While validating the implemented solution, other than the expected results (text and images), we observed some limitations in the used libraries: unexpected and irrelevant images were also extracted e.g. journal, publisher’s logos and header-footer images embedded inside document (e.g. images added by the publishers, to provide publishing details), text was not always in good rendering order, especially when there were text-based mathematical equations with super and subscripts; and in case of double or multicolumn PDF files, most of the libraries’ rendering order is not correct. During extracting text, we found that some important symbols were missed and spaces were generated for some paragraphs. We found that it was not possible to extract particular images that are created as a combination of different sub-images and text objects in the manuscript. In these cases, text is found in extracted text area and all extracted sub-images are image sections, with the possibility of missing some sub-images as well. Moreover, when we applied OCR to different images (extracted or loaded), we found that its performance does vary with respect to the complexity of inputted images. In case of special characters (e.g. Greek delta, alpha, beta etc.), it does not perform well unless these are hard wired in the software.

In comparison to earlier mentioned tools; MSL possess some advantages as well as limitations. For instance, Dolores help user in adding custom tags to the PDF document and create semantic model associated to the processed class of documents, PDF2HTML implements conditional random fields (CRF) based model to learn semantics from processed PDF page’s content, PDF-Analyzer devised a model based on rectangular objects for the analysis on PDF documents, XED applies method to combine PDF symbol analysis with traditional document image processing technique. MSL does not apply any of these methods and support such features. However, MSL does support segmentation of text, provides text in correct reading order, enable users with keywords based search and provide extraction of embedded text from figures (using OCR), which none of these tools does. To enhance the functionality of the MSL program (e.g. our standard version available here for download), we give a table of the most often used special symbols in biomedical literature ( Table 3). Depending on your application in mind, you thus simply extend the MSL parser by considering also these special characters occurring often in your texts.

**Table 3.** Special symbols found in biomedical literature ^1^.

Number	Special Symbols	Name
1	Δ	Delta
2	α	Alpha
3	β	Beta
4	ϕ	Phi

^1^The table illustrates that special characters occurring most often in the texts of choice enhance further MSL capabilities if incorporated in addition in the parser. This is, however, a text-dependent additional modification of the MSL program.

One in-house example is the DrumPID database ^39^, where different types of data and images are warehoused by us and an improved separation and retrieval of text versus figure legends, image descriptions etc. is highly useful and currently applied. The latest version of DrumPID allows understanding and screening of compounds for their effects in protein interaction networks. It is helpful in exploring potential antibiotic lead structures, studing individual pathways and potential targets in various organisms.

We compared ( Table 4) MSL with with different, earlier discussed related software applications: A-PDF, PDF Merge Split Extract, BePDF, KPDF, MuPDF, Xpdf tool, Power PDF, PDF2HTML, PDF-Analyzer, XED, Dolores and Layout-aware. The chosen and compared tools were picked based on the following criteria: 1) able to extract text from single, double and multiple column PDF files, 2) able to extract images from from single, double and multiple column PDF files, 3) provide options to search text and image based elements, 4) help in segmentation of text and images, 4) open source, 5) commercial, 6) freely available, 7) easily customizable, and 8) additionally, capable of analyzing embedded images with the application of OCR. There is not mathematical or statistical ranking of these tools were performed as most of these shared common features and obtained results were close. The reason to draw comparison is not at all to undermine the importance of any other available software applications but tried to justify the importance of MSL, as it’s the only one among which is fully open source, capable of extracting text and images from PDF files, applies OCR to get text from extracted chosen images, allow users to search, easily customizable (users can replace libraries) and freely available.

**Table 4.** Comparative analysis of MSL with different, earlier discussed related applications. This table compare different related applications (A-PDF, PDF Merge Split Extract, BePDF, KPDF, MuPDF, Xpdf tool, Power PDF, PDF2HTML, PDF-Analyzer, XED, Dolores, Layout-aware) to MSL. Comparison is drawn based on some major elements (Open source, PDF text extraction, PDF image extraction, OCR, Searching, IMRAD, Customizable and FREE).

Feature/Software	Open source	PDF text extraction	PDF image extraction	OCR	Searching	IMRAD	Customizable	FREE
A-PDF	No	Yes	Yes	No	No	No	No	No
PDF Merge Split Extract	No	Yes	Yes	No	No	No	No	No
BePDF	Yes	Yes	No	No	Yes	No	No	No
KPDF	Yes	Yes	No	No	Yes	No	No	Yes
MuPDF	Yes	Yes	Yes	No	Yes	No	No	Yes
Xpdf tool	Yes	Yes	Yes	No	No	No	No	Yes
Power PDF	No	Yes	Yes	No	Yes	No	No	No
PDF2HTML	No	Yes	No	No	No	No	No	No
PDF-Analyzer	No	Yes	No	No	No	No	No	No
XED	No	Yes	Yes	No	Yes	No	No	No
Dolores	No	Yes	Yes	No	Yes	No	No	No
Layout-aware	Yes	Yes	No	No	Yes	No	No	Yes
MSL	Yes	Yes	Yes	Yes	Yes	Yes	Yes	Yes

## Implementation & operation

MSL architecture is based on the Product Line Architecture (PLA) and Multi-Document Interface (MDI) developmental principles, and it is designed and developed (using C-Sharp programming language, Microsoft Dot NET Framework) following the key principles of *Butterfly* paradigm ^14, 38^. The work-flow of MSL is divided into two processes: (I) extraction and marginalization of text with respect to the division and placement of text in PDF file and keyword based search by using the iTextSharp, Bytescoute, Spire PDF libraries, and (II) extraction and analysis of figures by using the Spire PDF library and LEADTOOLS OCR.

It takes Portable Document Format (PDF) based literature files as input, performs partial physical structure analysis, and exports output in different formats e.g. text, images and XML files. It allows user to extract keywords and marginal (X and Y coordinates) information based text, have PDF file’s metadata information (title, author, creator, producer, subject, creation date, keywords, modified, number of pages and number of figures) and save extracted full and marginal text in text files.

Biomedical image extraction and analysis is one of the most complex tasks from the field of computer sciences and image analysis. Some of the mainstream approaches ^40– 45^ have been proposed towards the analysis of all kinds of images (e.g. flow charts, experimental images, models, geometrical shapes, graphs, image-of-thing, mix etc.). MSL allows user to automatically extracting images from the PDF files, let any selected image viewed via Windows default image viewer and apply implemented OCR. Other than extract images from PDF file, MSL allow user to load any image, apply OCR and export output in readable PDF file.

**Figure 5.**
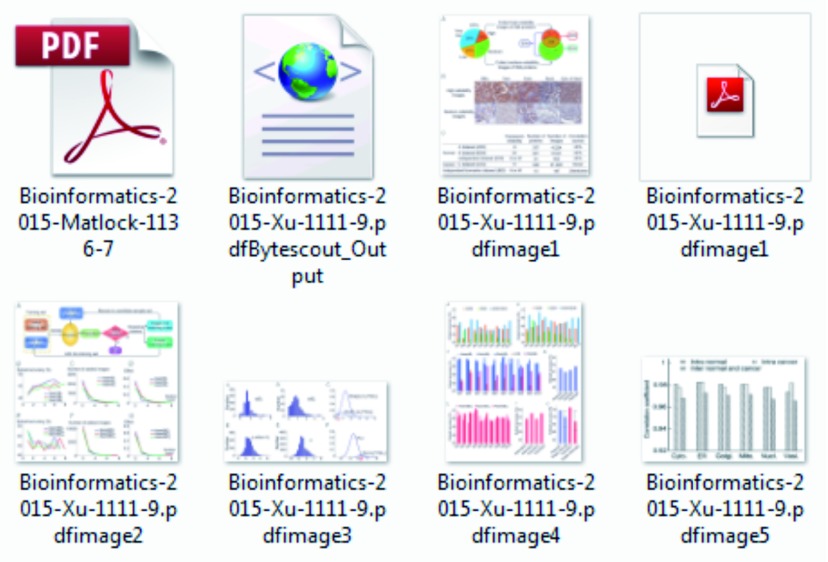
Screenshot of the all extracted images and generated files (XML and PDF). This figure shows different files generated during analysis of PDF document. PDF file (top, left) is the actual document, XML file is the structured (tagged) form of extracted text, second PDF file (top, right) is the extracted text from image (see Figure 3) and all other files are extracted image from PDF document.

MSL produces several out files in the parent folder including XML files (which include structured or tagged information), Images File (extracted from PDF file) and PDF files for all analyzed images using OCR ( Figure 5).

**Figure 6.**
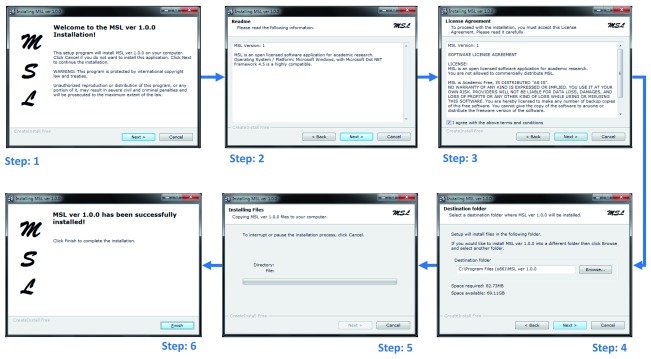
MSL six steps installation process.

MSL application is very simple to install and use. It was tested and can be well configured on a Microsoft Windows platform (preferred OS version: 7). MSL follows a simple six steps installation process ( Figure 6). After installation, it can be run by either clicking on the installed application’s icon at the desktop or execute application following sequence of steps: Start → All Programs → MSL 1.0.0 → MSL.

Regarding using the MSL application, one important point to remember is that it is based on different PDF text extraction, marginalization and figure extraction libraries, which are automatically configured during installation but used OCR by the LEADTOOLS is not a freely available library, which we have used upon academic research (free) license. The OCR library is also automatically configured during installation but its performance at different (non-licensed) machines is not confirmed. Moreover, the recommended display screen resolution size is 1680×1050 with landscape orientation.

## Conclusions

As most of the publishers are publishing in simple HTML and PDF formats, its not possible to segment and analyze raw form literature using available commercial and open-source software applications, as those are helpful in mainly text and image extraction. It will be helpful to have literature available in semantically tagged formats (e.g. XML, OWL etc.), so literature can be efficiently parsed, categorized and searched.

The development of a virtual research environment to store and link molecular data, can be well achieved and established if first the mixture of text, protocols and omics data is properly separated from images, figures and figure legends – a task for which our tool can be well suited. There are a number of databases (e.g. *Alzheimer’s Disease Neuroimaging Initiative (ADNI); Breast Cancer Digital Repository (BCDR); BiMed; Public Image Databases; Cancer Image Database (caIMAGE); COllaborative Informatics and Neuroimaging Suite (COINS); DrumPID; Digital Database for Screening Mammography (DDSM); Electron Microscopy Data Bank (EMDB); LONI image data archive; Mammography Image Databases (MID); New Database Provides Millions of Biomedical Images; Open Access Series of Imaging Studies (OASIS); Stanford Tissue Microarray Database (TMA); STRING; The Cancer Imaging Archive (TCIA); Whitney Imaging Center etc.*) which can directly profit from MSL by fast, automatic and rapid separation of text and text description from images and figure legends describing the images is important for further improvement of the database and its content.

The latest available and easy to use version of MSL has been tested and validated in-house. The advancements in information retrieval techniques for text and figure analysis combined with this sophisticated computational tool can support various studies.

## Data availability

The data referenced by this article are under copyright with the following copyright statement: Copyright: © 2018 Ahmed Z and Dandekar T

Data associated with the article are available under the terms of the Creative Commons Zero "No rights reserved" data waiver (CC0 1.0 Public domain dedication). 

*F1000Research*: Dataset 1. Extracted Images and Text from Papers tested using MSL, 10.5256/f1000research.7329.d108739 ^37^

## Software availability

### Software access

The software executable is freely available at the following web link: https://zenodo.org/record/30941#.Vi0PtmC5LHM

The software download section provides one executable: MSL, setup to be installed on the Microsoft Windows platform.

MSL has been NOT been developed for any commercial purposes but as a non-commercial prototype application for academic research, analysis and development purposes.

### Archived software files as at the time of publication

Mining Scientific Literature (MSL) Ver 1.0.0 (DOI: 10.5281/zenodo.30941).

### License

All associated files are licensed under the Academic Free License 3.0 (AFL 3.0).

## Supplementary material

**Supplementary Table S1. List of Papers (PDF files) tested using MSL.**

Supplementary which gives the list of some of those manuscripts from different publishers ( *F1000Research, PLOS, Hindawi, IEEE, BMC, PeerJ, Frontiers, ACM, Bentham Science and Oxford University Press*), which have been used for testing and validating the MSL application. The attached table provides the information about some of the extracted images and observed full and marginal text.
