# Supplementary material for: MSL: Facilitating automatic and physical analysis of published scientific literature in PDF format
Source: F1000Res. 2018 Apr 4;4:1453. Originally published 2015 Dec 16. [Version 3] doi: 10.12688/f1000research.7329.3 (PMC5897790; doi:10.12688/f1000research.7329.3)
Supplement: Supplementary file 2 [file f1000research-4-15700-s0001.tgz › 71295ce8-9f87-4ee7-9230-369bd3c6dc4c.pdf]

Table: List of Papers (PDF files) tested using MSL.

Supplementary Material: MSL: Facilitating automatic and physical analysis of published scientific literature in PDF format

Zeeshan Ahmed[1], Thomas Dandekar [2]

[1] The Jackson Laboratory for Genomic Medicine, Farmington 06032, CT, USA

[2] Department of Bioinformatics, Biocenter, University of Wuerzburg, Wuerzburg, 97074, Germany

Author notes:

Correspondence to: [a] zeeshan.ahmed@jax.org [b] dandekar@biozentrum.uni-wuerzburg.de

Supplementary Table: List of Papers (PDF files) tested using MSL.

The table gives the list of some of those manuscripts from different publishers (F1000Research, PLOS, Hindawi, IEEE, BMC, PeerJ, Frontiers, ACM, Bentham Science and Oxford University Press), which have been used for testing and validating the MSL application. This table provides the information about some of the extracted images and observed full and marginal text. All Extracted Images and Text (XML) are categorically provided in attached Supplementary Material: Raw Dataset.

Publishers	Extracted Text	Extracted Images
F1000-Reseasrch, (Ahmed et al., 2015) [1]	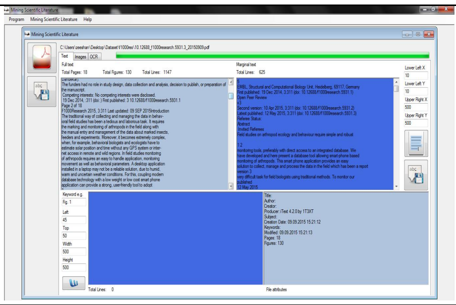	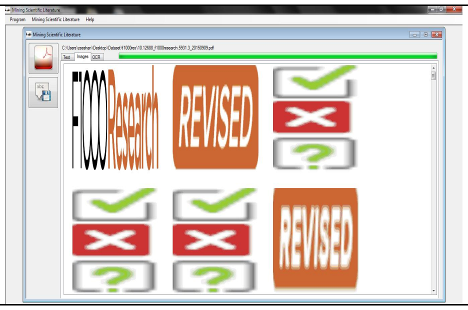

PLOS, (Pryszcz et al., 2015)[2]	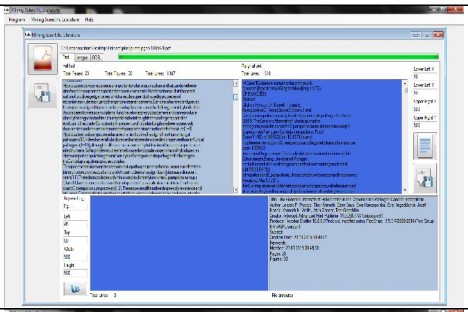	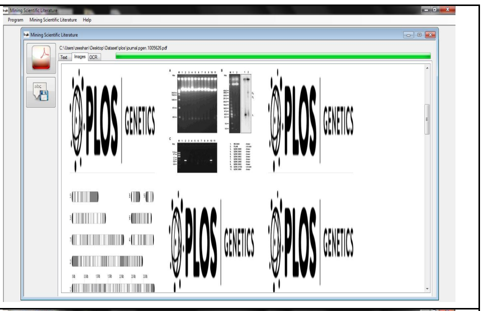
Hindawi, (Hernández et al., 2015)[3]	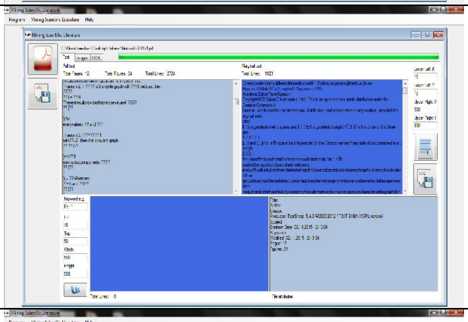	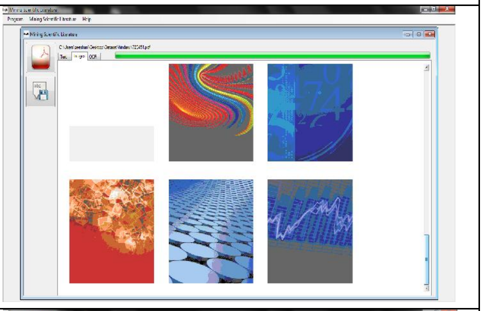
IEEE, (Ahmed and Gerhard, 2009)[4]	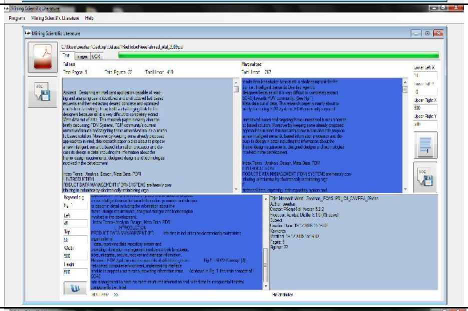	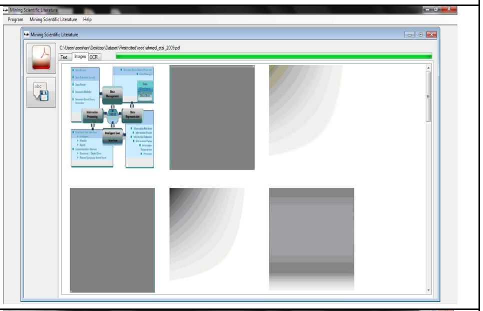
BMC, (Ahmed et al., 2013)[5]	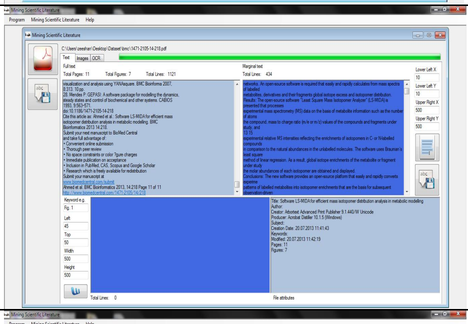	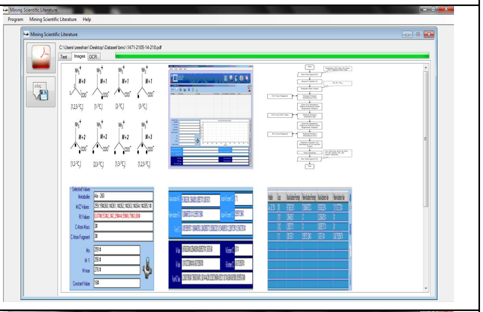
PeerJ, (Eren et al., 2015)[6]	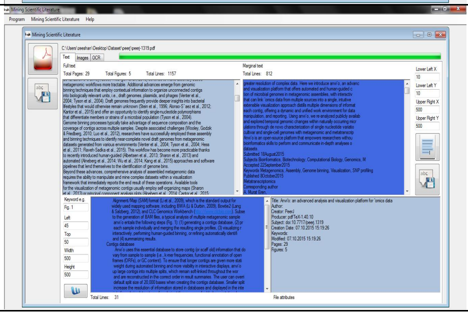	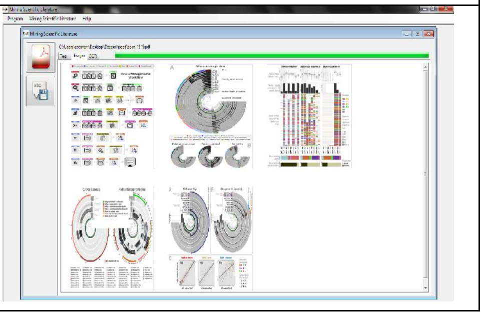

Frontiers, (Moreau et al., 2009) [7]	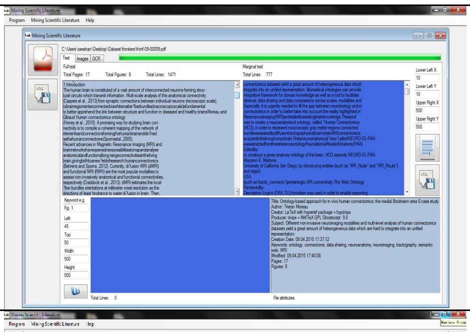	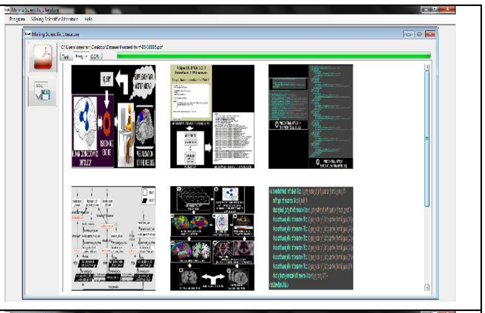
ACM, (Ahmed, 2009) [8]	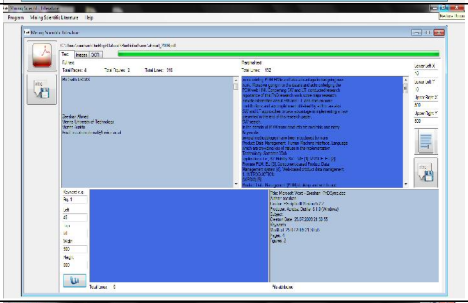	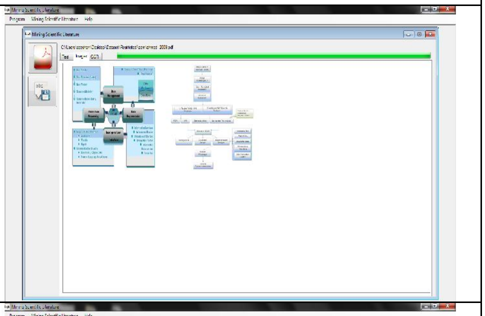
Bentham Science, (Ahmed and Helfrich-Förster, 2009) [9]	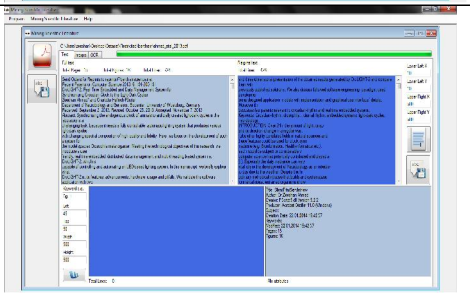	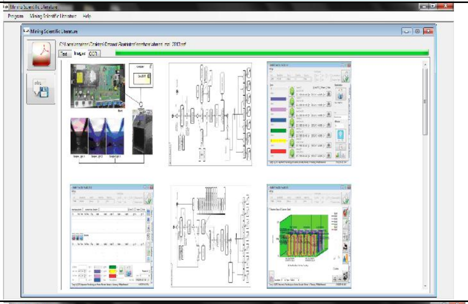
Oxford University Press, (Xu et al., 2015) [10]	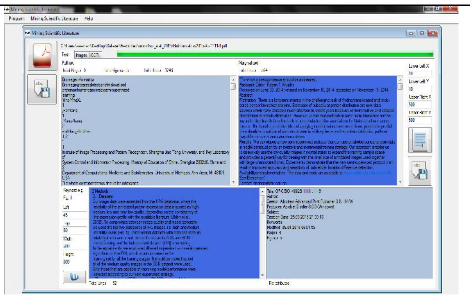	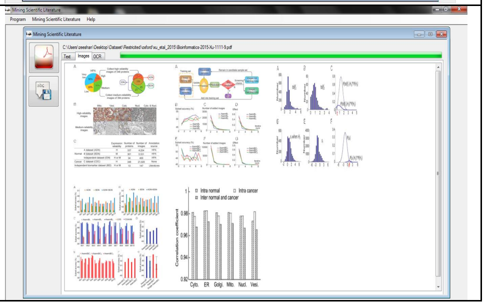

References

1. Ahmed Z, Zeeshan S, Fleischmann P *et al.* Ant-App-DB: a smart solution for monitoring arthropods activities, experimental data management and solar calculations without GPS in behavioral field studies [version 3; referees: 2 approved, 1 approved with reservations] *F1000Research* 2015, **3**:311 (doi: 10.12688/f1000research.5931.3)
2. Pryszcz LP, Németh T, Saus E *et al*. The Genomic Aftermath of Hybridization in the Opportunistic Pathogen Candida metapsilosis. *PLoS Genet* 2015, 11:10 (doi:10.1371/journal.pgen.1005626)

3. Hernández JC, Rodríguez JM, Sigarreta JM. Mathematical Properties of the Hyperbolicity of Circulant Networks. *Advances in Mathematical Physics* 2015, 11 (doi:10.1155/2015/723451)
4. Ahmed Z, Detlef G. Design implementation of I-SOAS IPM for advanced product data management. IEEE 2nd International Conference on Computer, Control and Communication, 2009.
5. Ahmed Z, Zeeshan S, Huber C, *et al*. Software LS-MIDA for efficient mass isotopomer distribution analysis in metabolic modelling. *BMC Bioinformatics* 2013, 14:218.
6. Eren AM, Esen ÖC, Quince C, *et al*. Anvi'o: an advanced analysis and visualization platform for 'omics data. *PeerJ* 2015, 3:e1319 (doi: 10.7717/peerj.1319)
7. Moreau T, Gibaud B. Ontology-based approach for in vivo human connectomics: the medial Brodmann area 6 case study. *Front. Neuroinform.* 2015, 9:9. (doi: 10.3389/fninf.2015.00009)
8. Ahmed Z. Intelligent semantic oriented agent based search (I-SOAS). In Proceedings of the 7th International Conference on Frontiers of Information Technology 2009. (doi: 10.1145/1838002.1838065)
9. Ahmed Z, Helfrich-Förster C. DroLIGHT-2: Real Time Embedded and Data Management System for Synchronizing Circadian Clock to the Light-Dark Cycles. *Recent Patents on Computer Science* 2013, 6:3.
10. Xu YY, Yang F, Zhang Y, Shen HB: Bioimaging-based detection of mislocalized proteins in human cancers by semi-supervised learning. *Bioinformatics*. 2015; 31(7):1111-9.
