# Supplementary material for: MSL: Facilitating automatic and physical analysis of published scientific literature in PDF format
Source: F1000Res. 2018 Apr 4;4:1453. Originally published 2015 Dec 16. [Version 3] doi: 10.12688/f1000research.7329.3 (PMC5897790; doi:10.12688/f1000research.7329.3)
Supplement: Peer review history

# Peer review: Referee response for version 3

**DOI:** 10.5256/f1000research.15700.r32753

**Published:** 2018-05-22

**Authors:** M. Julius Hossain

**Affiliations:** 1 Cell Biology and Biophysics Unit, European Molecular Biology Laboratory (EMBL), Heidelberg, Germany

The manuscript has been moderately improved by last two revisions. My main concern was that the work was not significantly new although it was claimed so. Authors tried to tone down their claim of novelty during the revisions. Authors added more comparisons and improved the readability of figures. While I am not fully convinced on the current version of the manuscript, I believe that the tool still could be useful for the community as it combines several features together.

I have read this submission. I believe that I have an appropriate level of expertise to confirm that it is of an acceptable scientific standard.

# Peer review: Referee response for version 3

**DOI:** 10.5256/f1000research.15700.r32754

**Published:** 2018-04-23

**Authors:** Karin Verspoor

**Affiliations:** 1 School of Computing and Information Systems, The University of Melbourne, Melbourne, VIC, Australia; 2 Health and Biomedical Informatics Centre, The University of Melbourne, Melbourne, VIC, Australia

Unfortunately I remain unconvinced about the contribution of the work presented in this manuscript. The results are presented through case studies rather than formal evaluation.

 Claims are made relating to various capabilities of the system that are not justified; for instance, the statement that the tool can split the text into "various IMRAD parts" is neither explained (in terms of Methodology -- does this simply involve searching for specific key words such as "Abstract" or "Introduction"?) nor directly evaluated. There are no quantitative evaluations of performance that support the claims that are made, only statements that the tool has been "validated in-house". This does not constitute a scientific evaluation of the tool. 

 Closer inspection of the Text box of Figure 1 suggests that the text is not in natural reading order; I see several lines from separate columns juxtaposed. The XML file produced for this text retains the columnar structure and shows

        The Lipid-Pro application can be configured on Microsoft Windows

 immediately followed by

         supported by Waters qTOF instrument platforms, acquires intact

 in the next column field without any straightforward way to extract the linear order of the text; the second sentence here clearly does not follow on from the first. I don't see how this format would be useful at all to anyone wanting to do natural language processing on this text, as the authors state. (NB: information retrieval and natural language processing are not the same and the textual representation assumed for NLP is very different than for IR; it is very important to retain the natural text flow and sentence structure for NLP tasks.)

 The premise of the work is still not adequately justified; the journals examined no longer (if they ever did; I doubt PLoS or PeerJ, for instance, ever did) publish work as images requiring OCR of the whole document. Lumping HTML and PDF together (as "simple formats") is clearly inappropriate; they have very different characteristics and indeed in HTML the text is nearly always in straightforward linear structure. However, not all PDFs are created equal and most PDFs have perfectly extractable (non-image/non-scanned) text these days through tools such as PDFbox. The authors have not responded to my prior comment that many publishers are already making documents available as nicely structured XML, in a format far preferable to what the authors are producing, due to richer provided semantic structure.

 The extraction and analysis of images themselves may be useful; doing OCR of text in an included image could be valuable. The authors claim that this is unique to their tool. It may be, and this may be the most useful part of their tool. However it is unclear how well the tool does this; a close look at Figure 1 identifies some clear errors, e.g. "Coiled" rather than "Collect"; "Nud" rather than "Nucl"; etc. What level of error is tolerable?

I have read this submission. I believe that I have an appropriate level of expertise to confirm that it is of an acceptable scientific standard, however I have significant reservations, as outlined above.

# Peer review: Referee response for version 3

**DOI:** 10.5256/f1000research.15700.r32751

**Published:** 2018-04-11

**Authors:** Juilee Thakar

**Affiliations:** 1 Department of Microbiology and Immunology, University of Rochester Medical Center, Rochester, NY, USA

I have no further comments.

I have read this submission. I believe that I have an appropriate level of expertise to confirm that it is of an acceptable scientific standard.

# Peer review: Referee response for version 2

**DOI:** 10.5256/f1000research.12318.r24964

**Published:** 2017-09-22

**Authors:** Karin Verspoor

**Affiliations:** 1 School of Computing and Information Systems, The University of Melbourne, Melbourne, VIC, Australia; 2 Health and Biomedical Informatics Centre, The University of Melbourne, Melbourne, VIC, Australia

This manuscript addresses the analysis of scientific articles published in PDF form, and introduces a software tool that essentially wraps a number of other tools to build a single tool that integrates a number of features.

 There is no novel methodological contribution in the tool itself that I could determine; the value is primarily in integrating the other tools into a user-facing tool. However, the value of a user-facing tool (as opposed to an automated tool that proceeds without human input) is not made clear or evaluated (are users happy to do the work expected of them?).

 As a very high level point on motivation, many scientific articles -- particularly in the open access literature which form the majority of the studied papers -- are already available as raw XML and it does not make sense to me to try to parse the PDF to infer structure (in a way that first requires user interaction, and second may introduce errors) that can be read directly from the publisher-produced XML. The PubMed Central repository is a repository of full-text, available online as structured HTML, and the open access collection which is downloadable in its entirety uses XML ( https://www.ncbi.nlm.nih.gov/pmc/tools/openftlist/). So in my opinion several of the key objectives of this tool are not needed for a large and growing proportion of the scientific literature. The argument could be made that older articles and articles from certain publishers are not available as "raw" XML, but the author neither make this argument nor specifically test this question. In addition, this XML resource provides a fantastic opportunity to automatically test (some aspects of) the performance of the authors tool. This is not done. The authors should consider the contribution of their tool in the context of such resources. The Introduction focuses on PubMed, but PubMed does not claim to search over the full-text literature (it is limited to abstracts by design), and PubMed Central is not mentioned here.

 There is one more tool that I am aware of -- and I suspect there are others -- that the authors do not mention; the Layout-Aware PDF tool ^1^. In general, I'd be interested to understand more deeply how the authors' tool differs from the various tools they mention, and why they selected the tools they did for their final system.

 The Results presented by the authors are not framed in any well-defined evaluation framework. While they mention "best overall performance" no indication of either specific quantitative or qualitative criteria used to assess the tool have been provided. What were the criteria that were used? Were there guidelines for how to "score" systems on various criteria? What is presented is largely ad hoc qualitative observations. On what basis was performance of the tool ranked?

**Comments on presentation:**

 It is poor practice to cite papers that are purely studied as artifacts -- a citation implies that you are referencing scientific content which you are not. Please remove citations to the papers that were used to test the system; they should be listed (preferably with DOIs) in Table 2, with a paper ID, and the paper IDs should be referenced in the article.

 There are a number of phrases in the manuscript that are awkward. "marginalization" does not mean "to find margins". Queries in PubMed are not "NLP quer[ies]" but rather *user* queries processed by an IR system (NB: arguably, IR systems don't even use NLP). The authors talk about "orthodox NLP approaches"; I have no idea what makes an NLP approach "orthodox". I suspect the authors mean "pipeline" rather than "product line". What is an "inherently multiplexed approach"?

 The long list of databases in the Conclusions doesn't add much to the manuscript; perhaps the example of the DrumPID should be pulled into a discussion section, and elaborated to clearly demonstrate the practical application and value of the tool.

I have read this submission. I believe that I have an appropriate level of expertise to confirm that it is of an acceptable scientific standard, however I have significant reservations, as outlined above.

## References

[1] RamakrishnanCPatniaAHovyEBurnsGA: Layout-aware text extraction from full-text PDF of scientific articles. .2012;7(1) : 10.1186/1751-0473-7-7 7 10.1186/1751-0473-7-7 22640904PMC3441580

# Response

**Published:** 2018-03-29

**Authors:** Zeeshan Ahmed

**Affiliations:** University of Connecticut Health Center, USA

**Reply: **Thank you so much for your recommendations.

*This manuscript addresses the analysis of scientific articles published in PDF form, and introduces a software tool that essentially wraps a number of other tools to build a single tool that integrates a number of features. There is no novel methodological contribution in the tool itself that I could determine; the value is primarily in integrating the other tools into a user-facing tool. However, the value of a user-facing tool (as opposed to an automated tool that proceeds without human input) is not made clear or evaluated (are users happy to do the work expected of them?). As a very high level point on motivation, many scientific articles -- particularly in the open access literature which form the majority of the studied papers -- are already available as raw XML and it does not make sense to me to try to parse the PDF to infer structure (in a way that first requires user interaction, and second may introduce errors) that can be read directly from the publisher-produced XML. The PubMed Central repository is a repository of full-text, available online as structured HTML, and the open access collection which is downloadable in its entirety uses XML (https://www.ncbi.nlm.nih.gov/pmc/tools/openftlist/).*

**Reply: **Thank you so much for your views.

*So in my opinion several of the key objectives of this tool are not needed for a large and growing proportion of the scientific literature. The argument could be made that older articles and articles from certain publishers are not available as "raw" XML, but the author neither make this argument nor specifically test this question. In addition, this XML resource provides a fantastic opportunity to automatically test (some aspects of) the performance of the authors tool. This is not done. The authors should consider the contribution of their tool in the context of such resources. The Introduction focuses on PubMed, but PubMed does not claim to search over the full-text literature (it is limited to abstracts by design), and PubMed Central is not mentioned here.*

**Reply: **We thank you so much for nice suggestion and have tried to revise manuscript accordingly.

*There is one more tool that I am aware of -- and I suspect there are others -- that the authors do not mention; the Layout-Aware PDF tool 1. In general, I'd be interested to understand more deeply how the authors' tool differs from the various tools they mention, and why they selected the tools they did for their final system.*

**Reply: **We thank you so much for nice suggestion and have tried to revise manuscript accordingly.

*The Results presented by the authors are not framed in any well-defined evaluation framework. While they mention "best overall performance" no indication of either specific quantitative or qualitative criteria used to assess the tool have been provided. What were the criteria that were used? Were there guidelines for how to "score" systems on various criteria? What is presented is largely ad hoc qualitative observations. On what basis was performance of the tool ranked?*

**Reply: **We thank you so much for nice suggestion and have tried to answer question in revision.

*It is poor practice to cite papers that are purely studied as artifacts -- a citation implies that you are referencing scientific content which you are not. Please remove citations to the papers that were used to test the system; they should be listed (preferably with DOIs) in Table 2, with a paper ID, and the paper IDs should be referenced in the article.*

**Reply: **We thank you so much for nice suggestion and have added DOIs.

*There are a number of phrases in the manuscript that are awkward. "marginalization" does not mean "to find margins". Queries in PubMed are not "NLP quer[ies]" but rather user queries processed by an IR system (NB: arguably, IR systems don't even use NLP). The authors talk about "orthodox NLP approaches"; I have no idea what makes an NLP approach "orthodox". I suspect the authors mean "pipeline" rather than "product line". What is an "inherently multiplexed approach"?*

**Reply: **We thank you so much for nice suggestion and have tried to revise manuscript accordingly.

*The long list of databases in the Conclusions doesn't add much to the manuscript; perhaps the example of the DrumPID should be pulled into a discussion section, and elaborated to clearly demonstrate the practical application and value of the tool.*

**Reply: **We thank you so much for nice suggestion and have added more details.

 We thank you so much for your time and excellent suggestions, which have helped us in improving the manuscript.

 With best wishes,

 Authors

# Peer review: Referee response for version 2

**DOI:** 10.5256/f1000research.12318.r24952

**Published:** 2017-09-06

**Authors:** Florencio Pazos

**Affiliations:** 1 Computational Systems Biology Group, National Center for Biotechnology (CNB-CSIC) (Spanish National Research Council), Madrid, Spain

The authors present a system for extracting the main text, the images, and the text within those (labels, etc), from scientific papers in PDF format. The system is implemented in an interactive desktop application for Windows and tested in 10 papers.

 Extracting the large amounts of scientific and medical information stored in an unstructured way is an important challenge. Any effort in that direction, such as that presented in this work, is of potential interest.

 My main concern with this work is that most of the features described are already available in existing software, even those described as “new”. For example, tools like “pdftotext” can parse multi-column PDFs, “pdfimages” extract the images within a PDF file, “OCRFeeder” detects the image elements and extract the texts, tables, etc, … On the contrary, some potentially newer features of MSL, such as recognizing the article parts (Abstract, References, … -page 3-) mentioned in Methods are not described later (Results). Problems of other tools mentioned in the Introduction, such as “removing irrelevant graphics” are not solved by MSL either.

 I think the comparison with other tools should be presented in terms of what these fail to detect while MSL does not, and vice versa. E.g. For a particular article “pdftotext” was not able to recognize the columns while MSL does, etc.

 In summary, better putting this system into the context of existing ones.

 The system is implemented as an interactive tool intended for analyzing a single article at a time, even presenting some of the final results (e.g. text extracted from images) back as PDF. For a single article (or a small number) human inspection will be better than any automated system. I guess the real potential of this system is in the automated parsing of large collections of articles. In my opinion the authors should focus the manuscript more on that.

I have read this submission. I believe that I have an appropriate level of expertise to confirm that it is of an acceptable scientific standard, however I have significant reservations, as outlined above.

# Response

**Published:** 2018-03-29

**Authors:** Zeeshan Ahmed

**Affiliations:** University of Connecticut Health Center, USA

**Reply: **Thank you so much for your recommendations.

*The authors present a system for extracting the main text, the images, and the text within those (labels, etc), from scientific papers in PDF format. The system is implemented in an interactive desktop application for Windows and tested in 10 papers. Extracting the large amounts of scientific and medical information stored in an unstructured way is an important challenge. Any effort in that direction, such as that presented in this work, is of potential interest.*

**Reply: **Thank you so much for your views and we agree with you.

*My main concern with this work is that most of the features described are already available in existing software, even those described as “new”. For example, tools like “pdftotext” can parse multi-column PDFs, “pdfimages” extract the images within a PDF file, “OCRFeeder” detects the image elements and extract the texts, tables, etc, …*

**Reply: **Thank you so much and we respect your points that many competing applications do exist but that still doesn't not negatively impact our contributions.

*On the contrary, some potentially newer features of MSL, such as recognizing the article parts (Abstract, References, … -page 3-) mentioned in Methods are not described later (Results).*

**Reply: **We thank for nice suggestion and have tried to revise manuscript accordingly.

*Problems of other tools mentioned in the Introduction, such as “removing irrelevant graphics” are not solved by MSL either.*

**Reply: **Thank you so much and we respect your point that it’s not a complete solution, as future research and development is also recommended but still helps at some good levels.

*I think the comparison with other tools should be presented in terms of what these fail to detect while MSL does not, and vice versa. E.g. For a particular article “pdftotext” was not able to recognize the columns while MSL does, etc.*

**Reply: **We thank for nice suggestion and have added comparison.

*In summary, better putting this system into the context of existing ones.*

**Reply: **We thank for nice suggestion and have tried to revise manuscript accordingly. 

*The system is implemented as an interactive tool intended for analyzing a single article at a time, even presenting some of the final results (e.g. text extracted from images) back as PDF. For a single article (or a small number) human inspection will be better than any automated system. I guess the real potential of this system is in the automated parsing of large collections of articles. In my opinion the authors should focus the manuscript more on that.*

**Reply: **We agree with you and thank so much for nice suggestion. We mainly tried to focus on that part but as one of the examples to show the strength of system, we have included that.

 We thank you so much for your time and excellent suggestions, which have helped us in improving the manuscript.

 With best wishes,

 Authors

# Peer review: Referee response for version 1

**DOI:** 10.5256/f1000research.7898.r14625

**Published:** 2016-08-09

**Authors:** M. Julius Hossain

**Affiliations:** 1 Cell Biology and Biophysics Unit, European Molecular Biology Laboratory (EMBL), Heidelberg, Germany

In this manuscript authors presented a computational tool that extracts text and images from PDF files. In general the manuscript is interesting considering that it can analyze various types of PDF files from different scientific areas based on the keywords and coordinates. However, it lacks technical novelty over the published literatures and needs additional input on the image analysis section before indexing.

 Extraction of texts and images from scientific publications has been presented in various domains: computer science ^1^, biomedical ^2^^-^^4^, chemistry ^5^, proteomics ^6^ and so on. The manuscript by Zeeshan Ahmed and Thomas Dandekar presents an incremental innovation without providing clear technological advancement in the field. The objective of performing both physical and logical structure analysis of all kinds of PDF files as mentioned in the manuscript has not been sufficiently supported by technological contribution described in Methods section.

 The image processing section the manuscript has been very brief. It does not provide any advanced image analysis technique as mentioned in the abstract. Authors should mention how exactly segmentation of figures and labels are performed and how they are represented to make logical connection between different entities in order to perform further analysis and customized visualization.

 The framework has been tested with a very small set of PDF files and no qualitative/quantitative result reporting the accuracy with respect to manually annotated files was presented. It would be good to increase the number test files and include the results of qualitative/quantitative analysis.

 Some of the figures (Figures 1, 3, 4 and 6) in the manuscript are hard to see the details in both online and print format. These figures could be reformatted.

I have read this submission. I believe that I have an appropriate level of expertise to confirm that it is of an acceptable scientific standard, however I have significant reservations, as outlined above.

## References

[1] ClarkCDivvalaS: Looking beyond text: Extracting figures, tables and captions from computer science papers. .2015; Reference source

[2] LopezLDYuJArighiCNHuangHShatkayHWuC: An Automatic System for Extracting Figures and Captions in Biomedical PDF Documents. .2011; 10.1109/BIBM.2011.26 578-581 10.1109/BIBM.2011.26 Reference source

[3] LopezLDYuJTudorCOArighiCNHongzhanHVijay-ShankerKWuK: Robust segmentation of biomedical figures for image-based document retrieval. .2012; 10.1109/BIBM.2012.6392706 1-6 10.1109/BIBM.2012.6392706 Reference source

[4] LopezLDYuJArighiCTudorCOToriiMHuangHVijay-ShankerKWuC: A framework for biomedical figure segmentation towards image-based document retrieval. .2013;7 Suppl 4: 10.1186/1752-0509-7-S4-S8 S8 10.1186/1752-0509-7-S4-S8 24565394PMC3856606

[5] ChoudhurySRMitraPKirkASzepSPellegrinoDJonesSGilesCL: Figure Metadata Extraction from Digital Documents. .2013; 10.1109/ICDAR.2013.34 135-139 10.1109/ICDAR.2013.34 Reference source

[6] KouZCohenWWMurphyRF: Extracting information from text and images for location proteomics. .2003;

# Peer review: Referee response for version 1

**DOI:** 10.5256/f1000research.7898.r11637

**Published:** 2016-01-13

**Authors:** Juilee Thakar

**Affiliations:** 1 Department of Microbiology and Immunology, University of Rochester Medical Center, Rochester, NY, USA

The manuscript titled “MSL: Facilitating automatic and physical analysis of published scientific literature in PDF format” addresses an important issue of extracting information from published manuscripts. However, the following issues must be clarified before indexing.

In the text mining section authors say that there is no tool to perform physical and logical structural analysis of PDF files. However, in the next paragraph they describe “Dolores” for logical structure analysis. Authors should describe how their method is different than Dolores.

Legends of all the figures should be more descriptive so that figures are understandable on their own. Each component of the figure should be described in the legend.

The results section is missing. Is it integrated in the discussion section? It is unclear what exactly the results were.

The article will be much clear if all the libraries (described on page 4 second paragraph) are described in the form of a table.

Authors should include a clear metric to estimate performance of the algorithm. This can be achieved by comparison with existing tools or through comparative analysis. A clear example showing the information extracted from several PDF files to address a biologically relevant example will be useful.

It is not clear whether the text extracted from the PDF files is actually coming from figure legends or related to the main body of the manuscript. Also, how is this text organized?

The authors mention that unexpected and irrelevant images were extracted. It is not clear how authors address that. It is absolutely essential to address that.

Minor corrections:

Page 2 second column: The definition of MSL is not the same as described in the abstract

I have read this submission. I believe that I have an appropriate level of expertise to confirm that it is of an acceptable scientific standard, however I have significant reservations, as outlined above.

# Response

**Published:** 2018-04-11

**Authors:** Juilee Thakar

**Affiliations:** University of Rochester, USA

Thanks for responding to my suggestions.
